# The Impact of Tuberculosis on the Well-Being of Adolescents and Young Adults

**DOI:** 10.3390/pathogens10121591

**Published:** 2021-12-08

**Authors:** Patricia Moscibrodzki, Leslie A. Enane, Graeme Hoddinott, Meredith B. Brooks, Virginia Byron, Jennifer Furin, James A. Seddon, Lily Meyersohn, Silvia S. Chiang

**Affiliations:** 1 Department of Clinical Research, London School of Hygiene and Tropical Medicine, London WC1E 7HT, UK; 2 The Ryan White Center for Pediatric Infectious Disease and Global Health, Department of Pediatrics, Indiana University School of Medicine, Indianapolis, IN 46202, USA; lenane@iu.edu; 3 Desmond Tutu TB Centre, Department of Paediatrics and Child Health, Stellenbosch University, Cape Town 8000, South Africa; graemeh@sun.ac.za (G.H.); james.seddon@imperial.ac.uk (J.A.S.); 4 Department of Global Health and Social Medicine, Harvard Medical School, Boston, MA 02115, USA; meredith_brooks@hms.harvard.edu (M.B.B.); virginia_byron@hms.harvard.edu (V.B.); jenniferfurin@gmail.com (J.F.); 5 Sentinel Project on Pediatric Drug-Resistant Tuberculosis, Boston, MA 02115, USA; 6 Department of Infectious Diseases, Imperial College London, London W2 1NY, UK; 7 Center for International Health Research, Rhode Island Hospital, Providence, RI 02903, USA; lmeyersohn@gmail.com (L.M.); silvia_chiang@brown.edu (S.S.C.); 8 Division of Pediatric Infectious Diseases, Department of Pediatrics, Alpert Medical School of Brown University, Providence, RI 02903, USA

**Keywords:** youth-friendly, differentiated service delivery, adherence, stigma, Zimbabwe

## Abstract

The health needs of adolescents and young adults (AYAs) have been neglected in tuberculosis (TB) care, control, and research. AYAs, who are distinct from younger children and older adults, undergo dynamic physical, psychological, emotional, cognitive, and social development. Five domains of adolescent well-being are crucial to a successful transition between childhood and adulthood: (1) Good health; (2) connectedness and contribution to society; (3) safety and a supportive environment; (4) learning, competence, education, skills, and employability; and (5) agency and resilience. This review summarizes the evidence of the impact of TB disease and treatment on these five domains of AYA well-being.

## 1. Introduction

An estimated 850,000 adolescents—defined by the World Health Organization (WHO) as individuals between 10–19 years of age—and an additional 1 million young adults—individuals between 20–24 years of age—become sick with tuberculosis (TB) disease each year [1,2]. The period between the ages of 10 and 24 years represents a critical period of biological growth and transitions in social roles [3]. During this period, individuals acquire the physical, cognitive, emotional, social, and economic resources that serve as the foundation for well-being for the rest of their lives. Five domains of well-being, as defined by a committee of adolescent health experts led by David Ross, are crucial for a successful transition from childhood to adulthood: (1) Good health; (2) connectedness and contribution to society; (3) safety and a supportive environment; (4) learning, competence, education, skills, and employability; and (5) agency and resilience (Table 1) [4]. TB disease and treatment can negatively impact these domains of well-being and thereby derail the growth and transitions that characterize this stage of life. 

Given the large number of adolescents and young adults (AYAs, ages 10–24 years) affected by TB and the importance of this developmental phase for acquiring the foundation for a healthy, productive adulthood, it is critical for healthcare providers, community leaders, and policymakers to understand how TB disease and treatment impact AYAs’ well-being. This review aims to highlight the effects of TB on AYA well-being, with the overarching goals of identifying urgent research gaps and informing policies and practices to address the TB epidemic among this cohort. 

## 2. Materials and Methods

We consulted three sources for this narrative review. First, we searched six databases—PubMed, PsycInfo, CINAHL, Global Health, Web of Science, and Google Scholar—for relevant studies using the following search terms (and variations): Adolescent, tuberculosis, behavioral symptoms, social stigma, social isolation, and well-being. Second, we included other published and unpublished data known to the authors. Third, we conducted outreach through the WHO’s Child and Adolescent TB Working Group to identify additional studies. 

We incorporated data from unpublished or ongoing studies conducted in Lima, Peru (principal investigator [PI]: Silvia S. Chiang) [5] and Harare, Zimbabwe (PI: Patricia Moscibrodzki) [6]. The methods of these studies are summarized in the Appendix A. Both studies were approved by the appropriate ethics committees.

## 3. The Impact of TB on AYA Well-Being

### 3.1. Domain 1: Good Health

Good health encompasses both physical and mental health. TB not only affects adolescents’ health during the illness period, but also may lead to long-term sequelae for physical and mental health. Infection with HIV and/or a drug-resistant strain of *M. tuberculosis* worsens these impacts. In this section, we examine how TB infection or disease impacts adolescents’ physical and mental health. 

#### 3.1.1. Rise in TB Infection and Disease

During adolescence and young adulthood, the incidence of TB disease increases dramatically, as evidenced by notification (i.e., case reporting) rates across settings [7,8,9,10]. The increase occurs earlier for females: In mid-adolescence (approximately ages 13–15 years), females have a higher notification rate, but by late adolescence, males have a higher notification rate.

Increased TB notification rates during adolescence and young adulthood likely stem from a combination of factors. First, the force of TB infection rises during this period [2,11]. Second, the age-dependent risk of progression from TB infection to disease—which is highest within the first five years of life and then decreases during the primary school years—increases in adolescence and peaks again between the ages of 20 and 29 years [12,13,14,15,16]. Cohort studies from the mid-20th century also demonstrated that this risk was highest for adolescent girls around the time of menarche [17,18]. The reasons underlying this increased risk of progression remain unclear, but likely stem from puberty-related changes in the immune response to *M. tuberculosis* [19,20]. 

Among AYAs, the most important risk factor for progression from TB infection to disease may be HIV co-infection. Globally, an estimated 1.7 million 10–19 year-olds and 3.4 million 15–24 year-olds are living with HIV [21]. Approximately one-third of new HIV infections globally occur in AYAs.^34^ In Africa, nearly 50% of the population is under 18 [22], and among 15–24 year-olds, women are more than twice as likely to have HIV infection than men [23].

A study in South Africa demonstrated that despite viral suppression, adolescents with perinatal HIV had a five-fold higher risk of TB disease compared to HIV-negative adolescents [24]. Meanwhile, those without viral suppression had a ten-fold higher risk of TB disease. Complex challenges for antiretroviral (ART) adherence during adolescence and young adulthood further contribute to vulnerability to TB among AYAs living with HIV [25].

Notably, despite AYAs’ increased risk of progression from TB infection to disease, most national TB programs do not prioritize this age group for tuberculosis preventive treatment (TPT), unless they are HIV-positive. Furthermore, even for HIV-positive AYAs, the degree of TPT uptake has seldom been reported [26]. 

#### 3.1.2. Clinical Presentation of TB 

While pulmonary TB in pre-pubertal children mostly presents as intrathoracic lymph node disease and its complications, post-pubertal AYAs tend to have adult-type pulmonary TB, which leads to inflammation and destruction of the lung parenchyma (frequently leading to lung cavitation) and is associated with more severe symptoms. Unlike intrathoracic lymph node disease, which is usually paucibacillary and therefore rarely infectious, adult-type pulmonary TB is generally highly infectious when untreated. 

Few data have been published on the frequency of extrapulmonary TB among AYAs. In cohort studies conducted in diverse settings, between 12% and 19% of adolescents had extrapulmonary TB only [27,28,29]. The most frequently reported types of extrapulmonary disease were pleural TB, cervical lymphadenitis, and TB meningitis [27,28,29].

HIV co-infection impacts the clinical manifestations of TB. Among adolescents in Ukraine, those with HIV co-infection had lower odds of lung cavitation compared to those without HIV; this pattern has also been observed in adults [8,30,31,32]. Viral suppression due to ART may shift the clinical presentation towards classic adult-type pulmonary TB [31]. In the Ukraine cohort, independent risk factors for extrathoracic disease were age 10–14 years (compared to age 15–19 years) and HIV co-infection [8].

#### 3.1.3. Reproductive Health

The risk of progression from TB infection to disease increases during pregnancy and the early post-partum period [33]. TB disease in pregnancy often presents with nonspecific symptoms and frequently affects extrapulmonary sites, particularly if the individual is also living with HIV. As a result, diagnosis and treatment may be delayed [34]^.^

AYAs with TB disease may be sexually active, have limited access to sexual and reproductive healthcare, and be at risk for pregnancy. Importantly, rifampicin and other rifamycins used to treat TB infection and disease reduce the efficacy of hormonal contraception. As a result, alternative contraceptive approaches should be explored with affected AYAs.

TB disease in pregnancy with or without HIV co-infection is associated with an increased risk of obstetric complications, including preeclampsia and miscarriage [33,35]. Infants whose mothers had TB disease during pregnancy are more likely to have TB disease in the first year of life, and may be at higher risk for low birthweight and death [33,35]. Given that adolescent pregnancy itself is associated with low birthweight, preterm delivery, preeclampsia, miscarriage, and stillbirth, pregnant adolescents with TB disease likely face an even higher risk of adverse outcomes [36]. To date, however, no published studies have examined the intersection between adolescent pregnancy and TB disease.

#### 3.1.4. Adverse Treatment Events 

First-line antituberculosis medications, including in combination with antiretrovirals, are generally well-tolerated in AYAs [37,38,39,40]. Elevated transaminases may be detected on routine monitoring labs, but levels usually normalize without treatment disruption or with the discontinuation of pyrazinamide [27,28,29]. Though rare, significant toxicities can occur, such as hepatitis in AYAs with significant alcohol consumption [37].

Few publications have reported the safety and tolerability of second-line TB medications for AYAs. In general, AYAs tolerate most second-line drugs better than older adults do. As compared to first-line therapy, however, second-line medications are associated with more frequent, diverse, and harmful adverse events. A few physical effects are particularly traumatizing for AYAs, as described in a qualitative study in Mumbai, India by Das et al. [41]. First, adolescents experienced physical pain and hearing loss from injectable agents. Second, study participants highlighted skin darkening due to clofazimine as one of the most stressful adverse events for patients and caregivers. Third, some second-line TB medications—cycloserine and, to a lesser extent, ethionamide—may have psychiatric effects. Infrequently, ethionamide, prothionamide, and para-amino salicylic acid (PAS) have been associated with hypothyroidism, which can lead to depression [42,43]. While data on adolescents with DR-TB and HIV co-infection are limited, a small study from Mumbai demonstrated that adverse treatment events in AYAs receiving both ART and second-line TB medications were frequent, with psychiatric events being particularly common [44].

#### 3.1.5. TB Treatment Outcomes and Adherence

In most published reports, ≥80% of AYAs are treated successfully for drug-susceptible TB. Most adolescents with unfavorable outcomes are lost to follow-up; <5% die [10,45]. An analysis of drug-susceptible TB treatment outcomes between 2004 and 2016 in South Africa demonstrated age-disaggregated case-fatality (CFRs) between 2.3 and 4.3 for adolescents; notably, the CFR peaked for boys in mid-adolescence and for girls in late adolescence [45]. In the same South African cohort, children and adolescents (0–19 years old) with HIV and TB had a CFR of 4.8 if they were receiving ART and 6.3 if they were not on ART; in comparison, children and adolescents without HIV had a CFR of 1.0 from TB [45]. Few studies have reported treatment outcomes for AYAs with drug-resistant (DR-TB), but the risks of death and loss to follow-up are greater than for drug-susceptible TB [2].

AYAs receiving treatment for TB disease are at risk for poor adherence, which encompasses both missed medication doses and loss to follow-up [46,47,48]. A few studies have analyzed programmatic data to identify predictors of poor adherence to drug-susceptible TB therapy [9,46,47,49,50,51]. Four risk factors—HIV coinfection [46,47,49,50], age 15–19 years [9,46,49], prior TB treatment [46,50], and male gender [46,51]—were observed in more than one setting. Qualitative data on adolescent adherence to TB treatment remain scarce but have been reported in a few studies. In multiple settings, family support is a key facilitator of adherence. Common reasons for a lack of family support include poor caregiver understanding about TB, severe poverty, family conflict or neglect, and older AYAs moving away from home [52]. AYAs are particularly sensitive to stigma, which is a central driver of adolescent disengagement from TB care [52,53]. Facility-based DOT exacerbates this barrier to adherence, as AYAs fear neighbors will see them receiving TB treatment. In addition, school and work schedule conflicts, travel, migration, side effects, and pill fatigue—particularly when in combination with antiretrovirals for HIV—were additionally mentioned barriers to adherence.

Chiang et al.’s work in Lima found that AYAs are at particularly high risk of poor adherence during the continuation phase, when symptoms generally have resolved and pill quantity and frequency decrease [5,51]. AYAs who were lost to follow-up cited symptom resolution, which led to the belief that treatment was no longer important. Moreover, at the end of the intensive phase (during which AYAs are instructed to isolate at home), AYAs eagerly return to school, work, and other activities, both because they feel better and because they no longer have to isolate at home. As a result, missed doses can occur because of schedule conflicts, distractions, and de-prioritization of treatment.

#### 3.1.6. Post-TB Sequelae

A substantial proportion of adults successfully treated for pulmonary TB live with post-TB lung disease (PTLD), a group of heterogeneous chronic respiratory abnormalities completely or partially attributable to previous TB [54]. Individuals with PTLD have abnormal pulmonary function testing and reduced respiratory capacity. To date, the risk of PTLD in adolescents has not been defined. Unlike adult lungs, adolescent lungs are still growing in volume and developing gas exchange capability, which may affect their vulnerability to PTLD [55].

AYAs tend to present late to care and have suboptimal treatment adherence; these factors may affect the risk of PTLD. Given the high number of AYAs who fall ill with TB each year, PTLD could be an important cause of chronic lung disease globally and could have significant implications for their ability to study, work, develop healthy exercise habits, and participate in sports and other leisure activities. Research is needed, however, to evaluate this hypothesis.

Extrapulmonary TB may also lead to long-term sequelae in AYAs. Neurological sequelae after TB meningitis include deficits in hearing, vision, language, speech, and movement [56,57]. Osteoarticular TB may lead to permanent deformities and reduced movement of the affected limb and/or joint.

#### 3.1.7. Effects of TB Illness and Treatment on Mental Health

TB and depression have been conceptualized as a syndemic: In adults, having TB disease is associated with depression, and depression increases the risk of TB reactivation and disease progression, as mediated through poverty, undernutrition, immunosuppression, and/or negative coping behaviors [58]. AYAs living in difficult socioeconomic circumstances are at increased risk for both mental health challenges and TB disease. AYAs with underlying or comorbid mental health conditions likely experience the negative impacts of TB disease and treatment even more profoundly.

Few studies have explored the intersection between TB and mental health among AYAs. Five of twenty-three (22%) adolescents with TB disease in Toronto, Canada demonstrated symptoms of depressed mood during TB treatment and were referred for psychiatric care [59]. In a study in Smolensk, Russia, 61% of adolescents on TB treatment experienced anxiety based on Taylor’s Manifest Anxiety Scale, compared to 35% of healthy adolescents without TB disease (*p* < 0.05) [60]. No published studies have measured the prevalence of depression among adolescents with TB disease.

Independent of underlying mental health challenges, the negative impacts of TB often lead to negative attitudes, depressive symptoms, behavioral changes, and psychosomatic symptoms [41,60]. Psychological trauma may relate to prolonged treatment courses, such as for DR-TB; pill burdens, particularly for DR-TB and/or HIV co-infection; pain from injectable medications or procedures; coping with adverse effects of treatment; and anxiety about their health. AYAs may experience irritability and sadness surrounding TB recurrences or treatment failure.

In interviews conducted by Chiang et al. in Lima, AYAs treated for drug-susceptible TB and their caregivers predominantly described their mood throughout their illness course as “down,” “little interest in things,” “depressed,” “bad,” and “sad” [5]. In a qualitative study conducted in Mumbai, India, Das et al. similarly reported that AYAs receiving treatment for DR-TB felt irritated and sad that they had TB, particularly if they had previous TB episodes and now had more complicated disease [41]. AYAs and family members interviewed by Das et al. felt traumatized, both physically and emotionally, by the intense treatment regimen. In the Western Cape, South Africa, Zimri et al. conducted a qualitative study using interviews and a body-mapping exercise to help AYAs articulate the psychosocial effects of treatment for multidrug-resistant TB (MDR-TB) [61]. AYAs described lying awake at night, worrying about the next day’s injections. Some drew needles on their body maps and explained that they felt scared that the needles were being injected “into [their] bones.” The authors concluded that these drawings reflected psychosocial effects—including depression—of treatment for MDR-TB that may be overlooked or neglected through routine clinical assessments and care.

The unmet psychosocial needs of AYAs with TB were discussed by TB care providers in in-depth interviews conducted in Zimbabwe by Moscibrodzki et al. Many providers highlighted that identifying mental health problems is insufficient to address the needs of AYAs, as age-appropriate mental health services are lacking [6].

Prolonged home isolation or hospitalization during TB treatment also contribute to mental health challenges for AYAs; these will be discussed in depth in Section 3.2.

### 3.2. Domain 2: Connectedness and Contribution to Society

Connectedness and contribution to society encompass opportunities to develop positive, meaningful relationships with others, including family and peers, and to be valued, respected, and accepted as part of the community. AYAs in some settings are isolated, either at home or in a hospital, for weeks or even months during TB therapy [62]. In this section, we examine the impact of isolation, as well as the effects of stigma, on AYAs’ feelings of connectedness and their mental health. We further examine the impact of TB—including the downstream effects of isolation and stigma—on three types of relationships important to AYAs’ experiences of connectedness, positive values, and contribution to society: Family, peer, and romantic/sexual partner relationships.

#### 3.2.1. Prolonged Home Isolation or Hospitalization

In most countries, infection control is accomplished through home isolation. In some settings, however, home isolation routinely lasts longer than the infectious period, which usually ends within two weeks of starting effective treatment [63]. Chiang et al. report that in Lima, AYAs routinely are instructed to isolate at home for a minimum of two months in order to document two negative sputum smears, as sputum samples are obtained only at the end of each treatment month [5]. Providers in many settings require two negative sputum smears to clear TB patients from isolation, but the operationalization of this requirement in Lima is problematic since the resolution of infectiousness typically occurs much sooner, and prolonged home isolation prevents AYAs from attending school, work, and social encounters. In countries of the former Soviet Union, children and adolescents with TB disease with any resistance pattern and of any anatomic site are routinely hospitalized for the entire duration of TB treatment, ostensibly to improve patient outcomes and limit TB transmission to the community. In other settings, such as in South Africa, children and adolescents may be hospitalized during the intensive phase of MDR-TB therapy, based on clinical judgement and an assessment of adherence feasibility in their home context.

Isolation and hospitalization may have more pronounced effects on AYAs than other age-groups. AYAs are profoundly sensitive to social exclusion, and the effects of isolation, stigma, and discrimination can have long-lasting consequences [2]. Chiang et al. observed that the social isolation required by TB treatment consistently left AYAs in Lima feeling low, sad, and depressed. Hospitalized AYAs with TB experience intense feelings of isolation, negative emotions, and symptoms of depression [64,65,66].

Further, in countries of the former Soviet Union, isolation wards for MDR-TB patients have been shown to worsen the effects of hospitalization on AYAs’ mental health. Patients in isolation wards are prohibited from leaving their rooms, whereas patients in general wards may interact with each other and receive visitors. In Karayeva et al.’s qualitative study in Kyiv City, Ukraine, AYAs cited feelings of loneliness, sadness, frustration, and disappointment while living in isolation wards, but described feeling better when transferred to general wards [65].

Both the cessation of normal day-to-day activities during these significant years, as well as long separations from family and friends, may potentially lead to short- and long-term social and emotional ramifications.

#### 3.2.2. Experiences of TB Stigma

AYAs may experience feelings of “being outcast” due to TB stigma in their communities. This stigma can be experienced in three ways: (1) The anticipation that someone will enact stigma toward oneself; (2) the experience of another person treating oneself differently and/or discriminating against them; and (3) a loss of self-esteem, fear, and/or shame. TB stigma associated with fear of TB transmission also often extends well beyond the infectious period and is intertwined with the stigma associated with other conditions, such as HIV infection, illicit drug use, migration, and poverty [41,52,65,66,67].

Below, we present several examples of stigma experienced by AYAs with TB [5,41,65,66]:

“*My neighbors knew I had TB, so they used to tell everyone that they should stay away from me, else I will spread the disease to them. I was asked not to sit outside home; they used to scold me that don’t come outside.*”

—15-year-old female, Mumbai, India

“*Wealthy people were there [at a tennis court in front of the hospital]. they disliked our company because we were poor. They humiliated us said unpleasant things.*”

—19-year-old female, Kyiv City, Ukraine

“*[The children in the neighborhood] call me names and they hit me and stuff like that. [They say] ‘TB thing, just go away.’ They think I’m going to infect them.*”

—12-year-old adolescent, Cape Town, South Africa

“*When I had tuberculosis, I felt that, as if no one wanted to get close to me, because they found out that I had the disease and they thought I was going to infect them. I felt like, as if I was something from another world, no one wants to get close to me.*”

—11-year-old female, Lima, Peru

#### 3.2.3. Impact of TB on Family Relationships

Stigma surrounds TB transmission, including within immediate and extended families [5,41]. Attribution or ‘responsibility’ for TB transmission may be a source of tension or reason for breakdown of family relationships. In Lima, many AYAs and their caregivers interviewed by Chiang et al. described the blame that permeated their family dynamics: For example, one side of the family blaming the other for TB [5]. Some AYAs also noted the burden of worrying about giving TB to a family member. In turn, AYAs and affected caregivers also internalized stigma, leading to intense feelings of shame or guilt [41].

Chiang et al. observed that when AYAs with TB disease received ambulatory treatment, avoiding transmission by the adolescent to other family members sometimes led to changes in the family’s domestic arrangements (e.g., who sleeps in which room in the home), and these changes, in turn, put a strain on family relationships. TB disease was also sometimes a source of shame within the family (e.g., a parent feeling ashamed that their child has TB), which sometimes caused extended family members to avoid or break contact with the TB-affected person [5]. One 17-year-old participant shared that he and his mother were forced out of his grandmother’s home, despite having completed three months of TB treatment. Another 15-year-old girl reported that her grandmother and maternal relatives gave her “*la espalda*”, or turned their back on her, prohibiting her from entering their house and serving her food on a plastic, disposable plate when they did begin to see her again.

The negative impacts of TB disease on family relationships were substantially magnified by hospitalization. Work by Karayeva et al. in Kyiv City shows that even when family members were allowed to visit, they were not able due to costs of transportation, among other barriers [65]. Given that many families could not afford the costs of telephonic or web-based call services, communication with family became limited. Family relationships were therefore interrupted, and post-hospitalization, these relationships often remained strained.

Of note, AYAs’ distance from family members can be particularly challenging for their mental health. Throughout the literature, it was clear that AYAs rely on family members for comfort when feeling down and reassurance that they will make it through their treatment. However, for many AYAs, this emotional support system is negatively impacted by hospitalization.

#### 3.2.4. Impact of TB on Peer Relationships

Upon diagnosis, AYAs across settings expressed concerns about what their peers would think. Most often they anticipated negative reactions and avoided disclosure. Some AYAs who did disclose their TB status to their peers shared that this anticipation of rejection was well-founded since they lost friends.

Not all AYAs who told their friends about their TB experienced rejection, however; on the contrary, some friends were supportive and non-judgmental. Even when isolated at home, several AYAs in Lima mentioned using social media platforms, such as Facebook and WhatsApp, to stay connected with friends [5]. AYAs hospitalized in Kyiv City also stayed in contact with their friends online [65].

Peer relationships can suffer not only from disclosure and rejection, but also from nondisclosure and the resulting isolation [5,64]. Some AYAs stopped participating in extracurricular activities with friends, particularly sports, because they were told by healthcare providers that it would worsen their illness, even after the intensive phase and isolation period [5]. One 17-year-old boy in Lima recalled the doctor who told him that he could no longer skate because he could “rupture” his lung if he fell. This warning frightened him, and he stopped skating for many months.

Remarkably, we found that in diverse settings, AYAs who were hospitalized for TB treatment formed strong, supportive relationships with each other. Karayeva et al. observed that among AYAs in Kyiv City who experienced prolonged hospitalizations for TB, being among other AYAs with TB afforded them peer support, education about TB, and even a respite from the pervasive stigma they felt in the community [65]. Relationships with other patients their own age led to more positive illness experiences overall. These conclusions were also supported in Franck’s finding in a study on TB stigma in Cape Town that hospital admission was sometimes cited as a *positive* social experience for children and young adolescents [66]. Likewise, in Tomsk, Russia, Zvonareva et al. report that hospitalized AYAs easily formed friendships with each other [64]. These strong, meaningful friendships often persisted beyond hospitalization.

#### 3.2.5. Impact of TB on Romantic/Sexual Partner Relationships

As with peer relationships, AYAs anticipated negative responses from romantic/sexual partners when disclosing TB disease. Most often, however, AYAs expressed that disclosure to partners was inevitable. While some AYAs reported that their partners were supportive, others experienced rejection [5]. In South Asia, a history of TB may harm one’s marriage prospects. In a qualitative study conducted in Nepal, Pakistan, and Bangladesh, Hatherall et al. reported that current or previous TB negatively impacts young people’s marriage prospects, particularly for women [68].

### 3.3. Domain 3: Safety and a Supportive Environment

AYA health and well-being are dependent on safety and supportive environments, which are inextricably linked with human rights [4]. Basic rights such as physical and emotional safety; food, water, hygiene, and shelter; and non-discrimination and equitable treatment are all necessary components of safe and supportive environments [4]. AYAs with TB are at risk of not having these rights fulfilled, as TB burdens are greatest in settings of poverty, inequality, and marginalization [69]. Being sick with TB further jeopardizes human rights.

#### 3.3.1. Income to Meet Basic Needs

AYAs and their families may experience major expenses from TB treatment, contributing to significant financial strain, which, in turn, impact AYAs’ ability not only to successfully complete treatment, but also to meet their basic needs [5,6,41,55,70]. Economic challenges associated with TB treatment can include travel costs to the hospital or facility and lost income when caregivers or other earners in the household are themselves ill with TB or need to attend to an ill AYA. Further, given the high energy expenditures of TB disease and the need for nutritious food, there can be added food expenses and related food insecurity [52,66].

Studies of AYAs with TB demonstrate that social and economic vulnerabilities further place AYAs at risk for poorer treatment outcomes, including loss to follow-up from treatment, treatment failure, and TB mortality [5,52,70,71,72]. Economic realities directly impact AYAs, who in some cases may leave treatment due to the need to work and earn income for their families [5,52]. Other AYAs skip doses because they do not have enough food to eat and do not tolerate the medicines on an empty stomach [6].

There is an urgent need to mitigate the economic impacts of TB on AYAs and families in order not only to preserve their human rights and access to basic needs, but also to facilitate successful treatment outcomes.

#### 3.3.2. Right to Privacy

Several aspects of TB care delivery interfere with AYAs’ right to privacy. For instance, having a separate clinic entrance and/or section for TB patients leads to inadvertent disclosure of their health status. In Lima, fear of being seen going through the TB entrance or in the TB section is a reason some AYAs disengage from treatment, particularly when they must visit the health center daily for DOT [5]. In Harare, AYAs fear that people will see them receiving pills at the health center and perceive that they are being treated for HIV [6].

Clofazimine-associated skin discoloration also may signal that an AYA is being treated for DR-TB [6,41]. This known adverse effect causes significant distress for AYAs and their families, who often are not aware that skin discoloration reverses soon after clofazimine is discontinued. In Das et al.’s study in Mumbai, parents expressed concern that their daughters’ skin discoloration from clofazimine will signal to the community that she has DR-TB, thus jeopardizing her marriage prospects [41].

#### 3.3.3. Right to the Highest Attainable Standard of Health

In qualitative studies, AYAs with TB and their family members describe experiences of discrimination in healthcare settings. Some AYAs recount poor treatment by clinic staff, which can discourage them from returning to the clinic and engaging in treatment [5,6]. Among AYAs who had undergone hospitalization, some describe disturbing effects of stigmatization and negative experiences with healthcare staff [65].

AYAs younger than 18 years of age are often excluded from TB treatment trials. Reasons for exclusion include the additional administrative burden of enrolling minors in research protocols and a lack of comfort engaging with this age group. As a result, younger AYAs may not be able to benefit from better treatments, such as shorter, injectable-free regimens for MDR-TB.

### 3.4. Domain 4: Learning, Competence, Education, Skills, and Employability

TB can impact the life and career trajectories of AYAs by disrupting education and employment [6]. Several factors—including the intensity and duration of TB treatment, the requirement in some settings to present to the health center for daily medication doses, and TB-associated stigma and discrimination—inhibit the ability of AYAs to continue schooling, including post-secondary education or vocational training, without disruption. The inverse also may be true: Educational and job commitments may pose barriers to TB treatment adherence.

#### 3.4.1. Absence from School or Vocational Training

Data on educational disruption in AYAs with TB are limited and, thus far, only qualitative in nature. Literature on other chronic diseases, however, has reported serious disruptions in schooling for AYAs [73,74,75,76,77,78].

Barriers to balancing TB treatment with schooling are myriad, and vary depending on age, developmental stage, disease status, and local context [5,6,65,66]. Clinic operating hours and long waiting times at overburdened health centers create a barrier to accessing care for AYAs, whose availability is dictated by class schedules. The cost of transportation to health centers may necessitate financial tradeoffs for older AYAs on a restrictive budget, as well as for family members accompanying younger AYAs to appointments. For AYAs with HIV-TB, separate clinics specializing in each condition create an added transportation and logistical burden to manage on top of educational responsibilities. AYAs may find it difficult to adjust their routines according to treatment schedules.

Prolonged home isolation or hospitalization exacerbates the problem of school absences during TB treatment. In Lima, several healthcare workers did not think that prolonged home isolation was problematic for AYAs’ education, since schools were required to provide educational support while students remained at home [5]. AYAs and their families, however, reported that this support never materialized. In settings where hospitalization for TB treatment is routine, TB hospitals may offer classes or tutoring for children and adolescents. Most AYAs in both Kyiv City and Cape Town, however, reported that these lessons were not as rigorous as normal schooling, while some AYAs hospitalized for treatment cited continued support from their community-based teachers as a critical factor in facilitating their return to the classroom [65,66].

#### 3.4.2. Educational Consequences of School Absences

AYAs with TB often need to repeat an academic semester or year due to school absences [5,6]. Students returning to school after a long absence face challenges in the classroom, such as skill regression and a difficult transition back to long school days [6]. In Kyiv City, students shared that skipping school for treatment made them do “terribly” and receive poor grades [65]. AYAs may also face the added challenge of physical and cognitive side effects from TB therapy that negatively affect school performance [41,60].

#### 3.4.3. Social Consequences of School Absences

As discussed in Section 3.2.2, AYAs with TB face stigma related to their disease status. Interviews conducted in Lima and Kyiv City found that many classmates and administrators were supportive of the AYAs with TB [5,65]. Yet, instances of discrimination and bullying still occur [5,65,66].

AYAs in Kyiv City and Cape Town reported difficulty reintegrating into a new cohort of classmates when required to repeat a grade due to TB-related absences. This led to added social difficulty that contributed to stigma in the school setting [65,66].

#### 3.4.4. Impact of TB on AYAs’ Career Trajectory and Future Earning Potential

Few studies have examined the impact of TB-related educational disruptions on AYAs’ future career trajectories. Of the 34 adolescents interviewed by Chiang et al. in Lima, 28 had been in secondary, post-secondary, or vocational school or military training at the time of their TB diagnosis [5]. Of those 28 adolescents, six dropped out of their program and, nearly a year later, still had not re-matriculated. Adolescents interviewed by Das et al. reported similar disruptions with schooling [41].

For older AYAs already in the workforce, the symptoms of TB, side effects of treatment, and the need to remain close to a TB treatment facility, have been reported to impact their ability to work. In Chiang et al.’s study in Lima, one 19-year-old female was forced to stop working because her job required her to climb stairs, which she was unable to do because of the weakness and fatigue she experienced from TB disease and treatment [5].

TB-related stigma also may impact AYAs’ future career trajectories. In a nationwide survey assessing the perceived impact of TB disease in Pakistan, 38% of adults interviewed felt that a diagnosis of TB would have a negative impact on one’s employability [79]. In Kyiv City, Karayeva et al. interviewed two AYAs who shifted their future plans because they were told their TB history would preclude them from pursuing their original career goals [65].

Further investigation is needed to better understand the impact of having TB disease on AYAs’ educational attainment, career trajectory, and future earning potential.

### 3.5. Domain 5: Agency and Resilience

TB may impact AYAs’ future trajectories by shaping their agency and resilience. “Agency” can be conceptualized as the ability to make sense of the environment, initiate change, make choices, and resist demands, while “resilience” refers to the potential for individuals to develop positively when exposed to adversity and stress [80,81]. We were unable to find data on the direct associations between TB and agency and resilience. However, mental health, TB-related stigma, relationships with peers and family members, and the ability to engage in school and work all have potential impacts on agency and resilience.

#### 3.5.1. Agency

Self-esteem is a key component of agency, and having TB is associated with low-self-esteem. Research in Lusaka, Zambia by Cremers et al. showed that 81.9% of TB patients, both children and adults, experienced any form of stigma, and 50.4% endorsed internalized stigma, which is associated with low self-esteem, shame, and feelings of inferiority [82]. Das et al.’s work in Mumbai further suggests that AYAs, more so than children, have long-lasting internalized stigma after DR-TB treatment [41]. Moreover, Moscibrodzki et al. reported that DOT for TB may hinder AYAs’ agency rather than strengthen it, as DOT can be perceived as restrictive and controlling [6].

#### 3.5.2. Resilience

Resilience is strongly associated with having rich social support networks and inversely associated with poor mental health and poor social support [83,84]. Therefore, TB may threaten AYAs’ resilience by interfering with their social networks and negatively impacting their mental health, as already discussed.

Nonetheless, some AYAs do demonstrate resilience in the face of their TB disease and treatment. In Kyiv City, Karayeva et al. asked TB survivors how their TB illness had changed their health and psychological outlook [65]. The responses were overwhelmingly positive—though it bears mentioning that this group of participants is highly self-selective, as half of the eligible AYAs identified for this study declined to participate, and some cited a desire to avoid revisiting their TB experience. A few AYAs interviewed by Karayeva et al. spoke about how their illness and hospitalization helped them develop healthier habits. Other AYAs in Kyiv City spoke about their diagnosis as a clarifying moment, helping them see what was important in life.

## 4. Conclusions

TB and its treatment have negative impacts across the domains of adolescent well-being defined by Ross et al. Further research is needed to better define and understand the challenges faced by AYAs with TB. Routine disaggregation of programmatic TB data for AYAs, stratified by five-year age bands, are also needed to identify the gaps and needs in TB care for this age group.

The primary limitation of this review is the dearth of published studies that explore the experiences and perspectives of AYAs affected by TB, despite the growing recognition of the risks and challenges faced by this group. For this reason, we have included unpublished data. The dataset from Chiang et al. also provides more insight into Latin America, a region that is underrepresented in the published literature [5]. Unfortunately, we were unable to identify any data, published or unpublished, from the Western Pacific and Southeast Asia, with the exception of the Indian subcontinent. Therefore, these high-TB burden areas are not represented in this review.

Despite the need for more data, there is evidence to support some immediate reforms to lessen the negative impacts of TB on AYAs. Because AYAs have an increased risk for progression from TB infection to disease, this age group should be prioritized in active case finding, contact tracing, and TPT. Additionally, because prolonged isolation and hospitalization particularly impact AYA mental health and disrupt education, work, and social relationships, such prolonged isolation measures should be minimized in consideration of the evidence for cessation of infectivity soon after initiation of effective treatment. For instance, some infection control guidelines recommend that patients be considered non-infectious once they have received adequate chemotherapy for two weeks, show clinical improvement, and have a negligible risk of MDR-TB [85]. For NTPs that prefer to document testing with two negative sputum smears, these samples could be obtained within the first two weeks of treatment, rather than waiting until the end of each treatment month, which would better balance the need for infection control and early release from isolation. Further, daily facility-based DOT disrupts schooling and contributes to stigma; alternative home-based TB treatment delivery approaches, which prioritize the needs of the patients and their families, should be developed, evaluated, and implemented for AYAs. NTPs should incorporate the WHO standards for adolescent-friendly services in order to provide quality care and optimize engagement and successful treatment of AYAs with TB. Finally, AYAs younger than 18 years of age should be included in TB treatment trials so that they can benefit from new advances in TB therapeutics. These and other reforms to provide AYA-friendly TB services are urgently needed, as the consequences of TB and its treatment can greatly impact AYA health outcomes, future livelihoods, and interpersonal relationships.

## Figures and Tables

**Table 1:** The five domains of adolescent well-being, as defined by Ross et al., and the ways in which they are impacted by TB disease and treatment [4].

Domain of Well-Being	Ways in Which TB Impacts Well-Being
Good health and optimum nutrition	AYAs, especially those with HIV, are at high risk of progression from TB infection to disease. Rifamycins decrease the efficacy of hormone-based contraception. Pregnant AYAs have an increased risk of progression from TB infection to disease and may be more likely to experience obstetric and neonatal complications. Adverse treatment events, such as skin darkening due to clofazimine and hearing loss from injectable agents, may be particularly traumatizing for AYAs. AYAs on TB treatment are at high risk of poor adherence, which may lead to treatment failure, relapse, increased resistance, and continued community transmission of *M. tuberculosis.* TB may adversely affect the long-term respiratory function of AYAs, whose lungs are still developing. TB disease and treatment may place AYAs at greater risk for mental health challenges.
Connectedness, positive values, and contribution to society	Prolonged home isolation or hospitalization for infection control purposes prevents AYAs from attending school, work, and social encounters. Both the cessation of normal day-to-day activities during these significant years, as well as long separations from family and friends, may potentially lead to short- and long-term social and emotional ramifications. TB-related stigma can negatively affect AYAs’ relationships with family, friends, and romantic partners, as well as their self-esteem.
Safety and a supportive environment	AYAs and their families may experience major expenses from TB treatment, contributing to significant financial strain, which, in turn, impact AYAs’ ability not only to successfully complete treatment, but also to meet their basic needs. Several aspects of TB care delivery interfere with AYAs’ right to privacy: for example, having a separate clinic entrance and/or section for TB patients leads to inadvertent disclosure of their health status. AYAs with TB may experience discrimination from health providers during their diagnostic evaluation and treatment.
Learning, competence, education, skills, and employability	Daily, facility-based DOT and prolonged isolation for infection control purposes interfere with AYAs’ education and vocational training. TB-related stigma can lead to experiences of discrimination at work and diminished job prospects.
Agency and resilience	Daily DOT for TB may hinder AYAs’ agency rather than strengthen it, as DOT can be perceived as restrictive and controlling. Resilience is strongly associated with having rich social support networks, and inversely associated with poor mental health and poor social support. Therefore, TB may threaten AYAs’ resilience by interfering with their social networks and negatively impacting their mental health.

## Data Availability

Unpublished data collected by P.M. and S.S.C. may be available upon request.

## Appendix A. Methodology of Unpublished Qualitative Studies

Lima, Peru

Chiang et al. conducted a qualitative study in Lima primarily to identify barriers and facilitators to adolescent adherence to TB treatment, and secondarily to explore the challenges faced by adolescents as a result of their TB disease and treatment [5]. Chiang et al. recruited three types of participants from public health centers run by the Ministry of Health: Adolescents aged 10–19 years who completed or were lost to follow-up from treatment for drug-susceptible pulmonary TB in the preceding 12 months, each adolescent’s primary caregiver, and health providers. The investigators enrolled 14 adolescents with good adherence, defined as treatment completion with <20% missed doses; 11 with suboptimal adherence, defined as treatment completion with ≥20% missed doses; and nine with loss to follow-up, defined as missing two or more consecutive months of treatment. The primary caregiver was the family member whom the adolescent identified as being the adult who provided the most support during TB treatment. Purposive sampling was used to maximize representation among adolescents with respect to adherence status and age. The healthcare provider group was a convenience sample of 15 nurses and nurse technicians with at least six months’ experience supervising TB treatment at a public health center.

Semi-structured, individual, in-depth interviews were conducted between July 2018 and June 2019 in Spanish by three trained Peruvian study workers. All interviews were conducted in private rooms, audio-recorded, and transcribed. The investigators conducted an applied thematic analysis of the interviews. Three investigators developed codes independently and finalized the codebooks through consensus. Five investigators independently coded all interviews. Eight (9.6%) interviews were double-coded to evaluate interrater agreement, which was ≥90% for all codes. Finally, three investigators reviewed the codes to identify emerging themes and select illustrative quotes, which were translated into English.

Harare, Zimbabwe

In Harare, Moscibrodzki et al. are conducting a qualitative study to explore the impact of TB on adolescents at the individual and societal levels to inform effective, adolescent-friendly care [6]. The first part of this study uses qualitative methods to explore the impact of TB diagnosis and treatment on the emotional and social welfare of patients in this age group. Semi-structured interviews—which began in April 2021—are being conducted with adolescents who are registered at seven primary health clinics in Harare; healthcare professionals, mostly TB nurses at the clinics; and policy stakeholders at the national and global levels. Interviews and workshops with adolescents are ongoing; and data analysis has not commenced, but raw transcripts were provided for inclusion within this literature review.

## References

[1] World Health Organization (2017). Global Accelerated Action for the Health of Adolescents (AA-HA!): Guidance to Support Country Implementation.

[2] Snow K.J., Cruz A.T., Seddon J.A., Ferrand R.A., Chiang S.S., Hughes J.A., Kampmann B., Graham S.M., Dodd P.J., Houben R.M. (2020). Adolescent Tuberculosis. Lancet Child Adolesc. Health.

[3] The Age of Adolescence—ScienceDirect. https://www.sciencedirect.com/science/article/abs/pii/S2352464218300221.

[4] Ross D.A., Hinton R., Melles-Brewer M., Engel D., Zeck W., Fagan L., Herat J., Phaladi G., Imbago-Jácome D., Anyona P. (2020). Adolescent Well-Being: A Definition and Conceptual Framework. J. Adolesc. Health.

[5] Chiang S.S. (2022). Teens on TB Treatment: Predicting Adherence through Clinical Decision Analysis. https://reporter.nih.gov/search/2-VGwyIr4km3Yy5sNwtauA/project-details/10136128.

[6] Moscibrodzki P. (2022). Exploring the Psychosocial Impact of Tuberculosis in Adolescents and Young People: A Qualitative Study in Zimbabwe.

[7] World Health Organization (2020). Global Tuberculosis Report 2020.

[8] Chiang S.S., Dolynska M., Rybak N.R., Cruz A.T., Aibana O., Sheremeta Y., Petrenko V., Mamotenko A., Terleieva I., Horsburgh C.R. (2020). Clinical Manifestations and Epidemiology of Adolescent Tuberculosis in Ukraine. ERJ Open Res..

[9] Kohlenberg A., Ködmön C., van den Boom M., van der Werf M.J. (2020). Tuberculosis Surveillance in Adolescents: What to Learn from European Union/European Economic Area Data?. Int. J. Tuberc. Lung Dis..

[10] Snow K., Yadav R., Denholm J., Sawyer S., Graham S. (2018). Tuberculosis among Children, Adolescents and Young Adults in the Philippines: A Surveillance Report. West. Pac. Surveill. Response J. WPSAR.

[11] Middelkoop K., Bekker L.-G., Liang H., Aquino L.D., Sebastian E., Myer L., Wood R. (2011). Force of Tuberculosis Infection among Adolescents in a High HIV and TB Prevalence Community: A Cross-Sectional Observation Study. BMC Infect. Dis..

[12] Marais B.J., Gie R.P., Schaaf H.S., Hesseling A.C., Obihara C.C., Starke J.J., Enarson D.A., Donald P.R., Beyers N. (2004). The Natural History of Childhood Intra-Thoracic Tuberculosis: A Critical Review of Literature from the Pre-Chemotherapy Era. Int. J. Tuberc. Lung Dis..

[13] Basu Roy R., Brandt N., Moodie N., Motlagh M., Rasanathan K., Seddon J.A., Detjen A.K., Kampmann B. (2016). Why the Convention on the Rights of the Child Must Become a Guiding Framework for the Realization of the Rights of Children Affected by Tuberculosis. BMC Int. Health Hum. Rights.

[14] Donald P.R. (2014). The North American Contribution to Our Knowledge of Childhood Tuberculosis and Its Epidemiology. Int. J. Tuberc. Lung Dis..

[15] Horsburgh C.R. (2004). Priorities for the Treatment of Latent Tuberculosis Infection in the United States. N. Engl. J. Med..

[16] The Risk of Tuberculosis in Children after Close Exposure: A Systematic Review and Individual-Participant Meta-Analysis—ScienceDirect. https://www.sciencedirect.com/science/article/abs/pii/S0140673620301665.

[17] (1972). Medical Research Council BCG and Vole Bacillus Vaccines in the Preventionof Tuberculosis in Adolescence and Early Adult Life. Bull. World Health Organ..

[18] Lincoln E.M. (1950). Course and Prognosis of Tuberculosis in Children. Am. J. Med..

[19] Seddon J.A., Chiang S.S., Esmail H., Coussens A.K. (2018). The Wonder Years: What Can Primary School Children Teach Us About Immunity to Mycobacterium Tuberculosis?. Front. Immunol..

[20] Baguma R., Mbandi S.K., Rodo M.J., Erasmus M., Day J., Makhethe L., de Kock M., van Rooyen M., Stone L., Bilek N. (2021). Inflammatory Determinants of Differential Tuberculosis Risk in Pre-Adolescent Children and Young Adults. Front. Immunol..

[21] Young People and HIV. https://www.unaids.org/en/resources/documents/2021/young-people-and-hiv.

[22] Children in Africa: Key Statistics on Child Survival and Population. https://data.unicef.org/resources/children-in-africa-child-survival-brochure/.

[23] Global HIV & AIDS Statistics—Fact Sheet. https://www.unaids.org/en/resources/fact-sheet.

[24] Frigati L.J., Wilkinson K.A., le Roux S., Brown K., Ruzive S., Githinji L., Petersen W., Belard S., Cotton M.F., Myer L. (2021). Tuberculosis Infection and Disease in South African Adolescents with Perinatally Acquired HIV on Antiretroviral Therapy: A Cohort Study. J. Int. AIDS Soc..

[25] Slogrove A.L., Mahy M., Armstrong A., Davies M.A. (2017). Living and Dying to Be Counted: What We Know about the Epidemiology of the Global Adolescent HIV Epidemic. J. Int. AIDS Soc..

[26] Kay A.W., Thivalapill N., Skinner D., Dube G.S., Dlamini N., Mzileni B., Fuentes P., Ustero P., Adams L.V., Mandalakas A.M. (2020). Predictors of Suboptimal Adherence to Isoniazid Preventive Therapy among Adolescents and Children Living with HIV. PLoS ONE.

[27] de Pontual L., Balu L., Ovetchkine P., Maury-Tisseron B., Lachassinne E., Cruaud P., Jeantils V., Valeyre D., Fain O., Gaudelus J. (2006). Tuberculosis in Adolescents: A French Retrospective Study of 52 Cases. Pediatr. Infect. Dis. J..

[28] Cruz A.T., Hwang K.M., Birnbaum G.D., Starke J.R. (2013). Adolescents with Tuberculosis: A Review of 145 Cases. Pediatr. Infect. Dis. J..

[29] Lotfian F., Bolursaz M.R., Khalilzadeh S., Baghaie N., Hassanzad M., Velayati A. (2016). Features of Adolescents Tuberculosis at a Referral TB’s Hospital in Tehran, Iran. Mediterr. J. Hematol. Infect. Dis..

[30] Padyana M., Bhat R.V., Dinesha M. (2012). HIV-Tuberculosis: A Study of Chest x-Ray Patterns in Relation to CD4 Count. N. Am. J. Med. Sci..

[31] Munthali L., Khan P.Y., Mwaungulu N.J., Chilongo F., Floyd S., Kayange M., Glynn J.R., French N., Crampin A.C. (2014). The Effect of HIV and Antiretroviral Therapy on Characteristics of Pulmonary Tuberculosis in Northern Malawi: A Cross-Sectional Study. BMC Infect. Dis..

[32] Kistan J., Laher F., Otwombe K., Panchia R., Mawaka N., Lebina L., Diacon A., Kana B., Martinson N. (2017). Pulmonary TB: Varying Radiological Presentations in Individuals with HIV in Soweto, South Africa. Trans. R. Soc. Trop. Med. Hyg..

[33] Mathad J.S., Gupta A. (2012). Tuberculosis in Pregnant and Postpartum Women: Epidemiology, Management, and Research Gaps. Clin. Infect. Dis..

[34] Kothari A., Mahadevan N., Girling J. (2006). Tuberculosis and Pregnancy—Results of a Study in a High Prevalence Area in London. Eur. J. Obstet. Gynecol. Reprod. Biol..

[35] Salazar-Austin N., Hoffmann J., Cohn S., Mashabela F., Waja Z., Lala S., Hoffmann C., Dooley K.E., Chaisson R.E., Martinson N. (2018). Poor Obstetric and Infant Outcomes in Human Immunodeficiency Virus-Infected Pregnant Women with Tuberculosis in South Africa: The Tshepiso Study. Clin. Infect. Dis..

[36] Leftwich H.K., Alves M.V. (2017). Adolescent Pregnancy. Pediatr. Clin. N. Am..

[37] Cruz A.T., Starke J.R. (2014). Safety and Completion of a 4-Month Course of Rifampicin for Latent Tuberculous Infection in Children. Int. J. Tuberc. Lung Dis..

[38] Villarino M.E., Scott N.A., Weis S.E., Weiner M., Conde M.B., Jones B., Nachman S., Oliveira R., Moro R.N., Shang N. (2015). Treatment for Preventing Tuberculosis in Children and Adolescents: A Randomized Clinical Trial of a 3-Month, 12-Dose Regimen of a Combination of Rifapentine and Isoniazid. JAMA Pediatr..

[39] Diallo T., Adjobimey M., Ruslami R., Trajman A., Sow O., Obeng Baah J., Marks G.B., Long R., Elwood K., Zielinski D. (2018). Safety and Side Effects of Rifampin versus Isoniazid in Children. N. Engl. J. Med..

[40] Jacobs T.G., Svensson E.M., Musiime V., Rojo P., Dooley K.E., McIlleron H., Aarnoutse R.E., Burger D.M., Turkova A., Colbers A. (2020). Pharmacokinetics of Antiretroviral and Tuberculosis Drugs in Children with HIV/TB Co-Infection: A Systematic Review. J. Antimicrob. Chemother..

[41] Das M., Mathur T., Ravi S., Meneguim A.C., Iyer A., Mansoor H., Kalon S., Hossain F.N., Acharya S., Ferlazzo G. (2021). Challenging Drug-Resistant TB Treatment Journey for Children, Adolescents and Their Care-Givers: A Qualitative Study. PLoS ONE.

[42] Garcia-Prats A.J., Schaaf H.S., Hesseling A.C. (2016). The Safety and Tolerability of the Second-Line Injectable Antituberculosis Drugs in Children. Expert Opin. Drug Saf..

[43] Thee S., Garcia-Prats A.J., Donald P.R., Hesseling A.C., Schaaf H.S. (2016). A Review of the Use of Ethionamide and Prothionamide in Childhood Tuberculosis. Tuberculosis.

[44] Isaakidis P., Paryani R., Khan S., Mansoor H., Manglani M., Valiyakath A., Saranchuk P., Furin J. (2013). Poor Outcomes in a Cohort of HIV-Infected Adolescents Undergoing Treatment for Multidrug-Resistant Tuberculosis in Mumbai, India. PLoS ONE.

[45] Osman M., du Preez K., Seddon J.A., Claassens M.M., Dunbar R., Dlamini S.S., Welte A., Naidoo P., Hesseling A.C. (2021). Mortality in South African Children and Adolescents Routinely Treated for Tuberculosis. Pediatrics.

[46] Mulongeni P., Hermans S., Caldwell J., Bekker L.-G., Wood R., Kaplan R. (2019). HIV Prevalence and Determinants of Loss-to-Follow-up in Adolescents and Young Adults with Tuberculosis in Cape Town. PLoS ONE.

[47] Enane L.A., Lowenthal E.D., Arscott-Mills T., Matlhare M., Smallcomb L.S., Kgwaadira B., Coffin S.E., Steenhoff A.P. (2016). Loss to Follow-up among Adolescents with Tuberculosis in Gaborone, Botswana. Int. J. Tuberc. Lung Dis..

[48] Guix-Comellas E.M., Rozas L., Velasco-Arnaiz E., Morín-Fraile V., Force-Sanmartín E., Noguera-Julian A. (2017). Adherence to Antituberculosis Drugs in Children and Adolescents in A Low-Endemic Setting: A Retrospective Series. Pediatr. Infect. Dis. J..

[49] Reif L.K., Rivera V., Bertrand R., Rouzier V., Kutscher E., Walsh K., Charles B., Pape J.W., Fitzgerald D.W., Koenig S.P. (2018). Outcomes across the Tuberculosis Care Continuum among Adolescents in Haiti. Public Health Action.

[50] de Oliveira M.C., Sant’Anna C.C., Luiz R.R., Kritski A.L. (2020). Unfavorable Outcomes in Tuberculosis: Multidimensional Factors among Adolescents in Rio de Janeiro, Brazil. Am. J. Trop. Med. Hyg..

[51] Chiang S.S., Dara M. (2020). TB in Children and Adolescents. Int. J. Tuberc. Lung Dis..

[52] Enane L.A., Eby J., Arscott-Mills T., Argabright S., Caiphus C., Kgwaadira B., Steenhoff A.P., Lowenthal E.D. (2020). TB and TB-HIV Care for Adolescents and Young Adults. Int. J. Tuberc. Lung Dis..

[53] Laycock K.M., Eby J., Arscott-Mills T., Argabright S., Caiphus C., Kgwaadira B., Lowenthal E.D., Steenhoff A.P., Enane L.A. (2021). Towards Quality Adolescent-Friendly Services in TB Care. Int. J. Tuberc. Lung Dis..

[54] Allwood B.W., Amaral A.F.S., van der Zalm M.M., Byrne A., Datta S., Egere U., Evans C.A., Evans D., Gray D.M., Hoddinott G. (2020). Post-Tuberculosis Lung Health: Perspectives from the First International Symposium. Int. J. Tuberc. Lung Dis..

[55] Neve V., Girard F., Flahault A., Boulé M. (2002). Lung and Thorax Development during Adolescence: Relationship with Pubertal Status. Eur. Respir. J..

[56] Duque-Silva A., Hampole V., Cheng Y.N., Flood J., Barry P.M. (2019). Outcomes of Pediatric Central Nervous System Tuberculosis in California, 1993–2011. J. Pediatric Infect. Dis. Soc..

[57] Chiang S.S., Khan F.A., Milstein M.B., Tolman A.W., Benedetti A., Starke J.R., Becerra M.C. (2014). Treatment Outcomes of Childhood Tuberculous Meningitis: A Systematic Review and Meta-Analysis. Lancet Infect. Dis..

[58] Sweetland A.C., Kritski A., Oquendo M.A. (2017). Addressing the Tuberculosis-Depression Syndemic to End the Tuberculosis Epidemic. Int. J. Tuberc. Lung Dis..

[59] Kam A., Ford-Jones L., Malloy P., Khan K., Kitai I. (2007). Active Tuberculosis among Adolescents in Toronto, Canada: Clinical Features and Delays in Diagnosis. Pediatr. Infect. Dis. J..

[60] Avdeeva T., Otvagin I., Myakisheva T., Rashkevich E. (2012). Tuberculosis in Adolescents and Young Patients in High Prevalence Region. Eur. J. Microbiol. Immunol..

[61] Zimri K., Casper R., Hoddinott G., Schaaf H.S., Garcia-Prats A.J., Rose P.C., Hesseling A.C., Viljoen L. (2020). A Novel Approach for Eliciting Adolescent MDR-TB Treatment Tolerability: Qualitative Data from South Africa. Int. J. Tuberc. Lung Dis..

[62] Seddon J.A., Shingadia D. (2014). Epidemiology and Disease Burden of Tuberculosis in Children: A Global Perspective. Infect. Drug Resist..

[63] Dharmadhikari A.S., Mphahlele M., Venter K., Stoltz A., Mathebula R., Masotla T., van der Walt M., Pagano M., Jensen P., Nardell E. (2014). Rapid Impact of Effective Treatment on Transmission of Multidrug-Resistant Tuberculosis. Int. J. Tuberc. Lung Dis..

[64] Zvonareva O., Witte S., Kabanets N., Filinyuk O. (2021). Adolescents in a Tuberculosis Hospital: Qualitative Study of How Relationships with Doctors, Caregivers, and Peers Mediate Their Mental Wellbeing. PLoS ONE.

[65] Karayeva E. (2020). The Impact of Hospitalization on Ukrainian Adolescents Who Have Completed Tuberculosis Treatment in Kyiv City, Ukraine. Master’s Thesis.

[66] Franck C. (2012). Assessing the Importance of Stigma in Children’s Experience of MDR-TB Treatment in the Western Cape Province, South Africa. Master’s Thesis.

[67] Kavanagh M.M., Gostin L.O., Stephens J. (2020). Tuberculosis, Human Rights, and Law Reform: Addressing the Lack of Progress in the Global Tuberculosis Response. PLoS ONE.

[68] Hatherall B., Newell J.N., Emmel N., Baral S.C., Khan M.A. (2019). “Who Will Marry a Diseased Girl?” Marriage, Gender, and Tuberculosis Stigma in Asia. Qual. Health Res..

[69] Citro B., Lyon E., Mankad M., Pandey K.R., Gianella C. (2016). Developing a Human Rights-Based Approach to Tuberculosis. Health Hum. Rights.

[70] Enane L.A., Davies M.A., Leroy V., Edmonds A., Apondi E., Adedimeji A., Vreeman R.C. (2018). Traversing the Cascade: Urgent Research Priorities for Implementing the “treat All” Strategy for Children and Adolescents Living with HIV in Sub-Saharan Africa. J. Virus Erad..

[71] Enane L.A., Apondi E., Omollo M., Toromo J.J., Bakari S., Aluoch J., Morris C., Kantor R., Braitstein P., Fortenberry J.D. (2021). “I Just Keep Quiet about It and Act as If Everything Is Alright”—The Cascade from Trauma to Disengagement among Adolescents Living with HIV in Western Kenya. J. Int. AIDS Soc..

[72] Enane L.A., Christenson J.C. (2021). Global Emerging Resistance in Pediatric Infections with TB, HIV, and Gram-Negative Pathogens. Paediatr. Int. Child Health.

[73] Merville O., Puangmala P., Suksawas P. (2021). School Trajectory Disruption among Adolescents Living with Perinatal HIV Receiving Antiretroviral Treatments: A Case-Control Study in Thailand. BMC Public Health.

[74] Zinyemba P., Pavlova M., Groot W. (2020). Effects of HIV/AIDS on Children’s Educational Attainment: A Systematic Literature Review. J. Econ. Surv..

[75] Vetsch J., Wakefield C.E., McGill B.C. (2018). Educational and Vocational Goal Disruption in Adolescent and Young Adult Cancer Survivors. Psycho-Oncology.

[76] Docherty S.L., Kayle M., Maslow G.R., Santacroce S.J. (2015). The Adolescent and Young Adult with Cancer: A Developmental Life Course Perspective. Semin. Oncol. Nurs..

[77] Sisk B.A., Fasciano K., Block S.D., Mack J.W. (2020). Impact of Cancer on School, Work, and Financial Independence among Adolescents and Young Adults. Cancer.

[78] Donnan B., Webster T., Wakefield C.E., Dalla-Pozza L., Alvaro F., Lavoipierre J., Marshall G.M. (2015). What About School? Educational Challenges for Children and Adolescents With Cancer. Educ. Dev. Psychol..

[79] Gilani S.I., Khurram M. (2012). Perception of Tuberculosis in Pakistan: Findings of a Nation-Wide Survey. J. Pak. Med. Assoc..

[80] De Mol J., D’Alcantara A., Cresti B. (2018). Agency of Depressed Adolescents: Embodiment and Social Representations. Int. J. Qual. Stud. Health Well-Being.

[81] Cordovil C., Crujo M., Vilariça P., Da Silva P.C. (2011). Resiliência em crianças e adolescentes institucionalizados [Resilience in institutionalized children and adolescents]. Acta Med. Port..

[82] Cremers A.L., de Laat M.M., Kapata N., Gerrets R., Klipstein-Grobusch K., Grobusch M.P. (2015). Assessing the Consequences of Stigma for Tuberculosis Patients in Urban Zambia. PLoS ONE.

[83] Färber F., Rosendahl J. (2018). The Association between Resilience and Mental Health in the Somatically Ill: A Systematic Review and Meta-Analysis. Dtsch. Ärzteblatt Int..

[84] Ozbay F., Johnson D.C., Dimoulas E., Morgan C.A., Charney D., Southwick S. (2007). Social Support and Resilience to Stress: From Neurobiology to Clinical Practice. Psychiatry.

[85] Migliori G.B., Nardell E., Yedilbayev A., D’Ambrosio L., Centis R., Tadolini M., van den Boom M., Ehsani S., Sotgiu G., Dara M. (2019). Reducing Tuberculosis Transmission: A Consensus Document from the World Health Organization Regional Office for Europe. Eur. Respir. J..

